# A Bayesian Treatment of the German Tank Problem

**DOI:** 10.1007/s00283-023-10274-6

**Published:** 2023-06-13

**Authors:** Cory M. Simon

**Affiliations:** School of Chemical, Biological, and Environmental Engineering, Oregon State University, Corvallis, OR USA

The German tank problem has an interesting historical background and is an engaging problem of statistical estimation for the classroom. The objective is to estimate the size of a population of tanks inscribed with sequential serial numbers, from a random sample. In this tutorial article, we outline the Bayesian approach to the German tank problem, whose solution assigns a probability to each tank population size, thereby quantifying uncertainty, and which provides an opportunity to incorporate prior information and/or beliefs about the tank population size into the solution. We illustrate with an example. Finally, we survey problems in other contexts that resemble the German tank problem.

## Background

To inform their military strategy during World War II (1939–1945), the Allies sought to estimate Germany’s rate of production and capacity of various types of military equipment (tanks, tires, rockets, etc.). Conventional methods to estimate armament production, including extrapolating data on prewar manufacturing capabilities, obtaining reports from secret sources, and interrogating prisoners of war, were mostly unreliable or contradictory.

In 1943, British and American economic intelligence agencies exploited a German manufacturing practice in order to statistically estimate their armament production. Specifically, Germany marked their military equipment with serial numbers as well as codes for the date and/or place of manufacture. Their intention was to facilitate the handling of spare parts and to trace defective equipment and parts back to the manufacturer for quality control. However, these serial numbers and codes on a captured sample of German equipment conveyed information to the Allies about Germany’s production.

To estimate Germany’s rate of production of tanks, the Allies collected serial numbers on the chassis, engines, gearboxes, and bogie wheels of samples of tanks by inspecting captured tanks and examining captured records.^1^ Despite lacking an exhaustive sample, the sequential nature of these serial numbers and patterns in the samples enabled the Allies to estimate Germany’s tank production. Postwar research has shown that serial number analysis gave more accurate estimates than the overestimates produced by conventional intelligence methods (Table 1).^2^ See Richard Ruggles and Henry Brodie’s article [44] for a detailed historical account of the serial number analysis used to estimate German armament production during World War II.

**Table 1:** Monthly production rate of tanks by Germany [44].

Month	Estimates		German records
	Conventional American & British Intelligence	Serial number analysis	
June, 1940	1000	169	122
June, 1941	1550	244	271
August, 1942	1550	327	342

### The German Tank Problem

Simplification of the historical context in which German tank production was estimated via serial number analysis [44] motivated the formulation of the textbook-friendly *German tank problem* [21]:

#### Problem statement.

During World War II, the German military is equipped with *n* tanks. Each tank is inscribed with a unique serial number in the set $\{1, \ldots , n\}$.

As the Allies, we do not know *n*, but we have captured (without replacement, of course) a sample of *k* German tanks with (ordered) inscribed serial numbers $(s_1, \ldots , s_k)$.
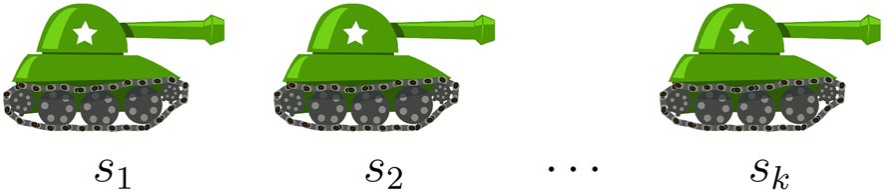


Assuming that every tank in the population was equally likely to be captured and *n* is fixed, our objective is to estimate *n* in light of the data $(s_1, \ldots , s_k)$.

In 1942, in a crowded restaurant in Washington, D.C., Alan Turing and Andrew Gleason discussed a variant of the German tank problem: “how to estimate the total number of taxicabs in a town after having seen a random selection of their license numbers” [13, 24]. Today, with its interesting historical background [44], the German tank problem is still a suitable dinner conversation topic and serves as an intellectually engaging, challenging, and enjoyable problem to illustrate combinatorics and statistical estimation in the classroom [3, 15, 27, 33].

**Uncertainty quantification.** Any estimate of the tank population size $n$ from the data $(s_1, \ldots , s_k)$ is subject to uncertainty, since we (presumably) have not captured all of the tanks (i.e., $k\ne n$, probably). Quantifying uncertainty in our estimate of *n* is important because high-stakes military decisions may be made on the basis of it.

**Our contribution.** In this pedagogical article, we outline the Bayesian approach to the German tank problem, whose solution assigns a probability to each tank population size, thereby quantifying uncertainty, and which provides an opportunity to incorporate prior information and/or beliefs about the tank population size into the solution.

### Survey of Previous Work on the German Tank Problem

**The frequentist approach.** Kim Border [7] calls the German tank problem a “weird case” in frequentist estimation. The maximum likelihood estimator of the tank population size *n* is the maximum serial number observed among the *k* captured tanks, $m^{(k)}:=\max _{i \in \{1, \ldots , k\}} s_i$. This is a biased estimator, since certainly $m^{(k)} \le n$.

Leo Goodman [21, 22] derives the minimum-variance unbiased estimator of the tank population size

1 $$\begin{aligned} {\hat{n}} = m^{(k)} + \left( \frac{m^{(k)}}{k}-1 \right) . \end{aligned}$$

To intuit ${\hat{n}}$, note that *n* must be greater than or equal to $m^{(k)}$, and if we observe large (small) gaps between the serial numbers $(s_1, \ldots , s_k)$ after sorting them (including the gap preceding the smallest serial number), then *n* is likely (unlikely) to be much greater than $m^{(k)}$. The estimator of *n* in (1) quantifies how far beyond the maximum serial number $m^{(k)}$ we should estimate the tank population size, based on the gaps; $m^{(k)}/k-1$ is the average size of the gaps. Goodman [21] also derives a frequentist two-sided $1-a$ confidence interval $m^{(k)}\le n \le x$ for *n*, where *x* is the greatest integer satisfying $\left( m^{(k)}-1\right) _{k} /(x)_k \ge a$ (the notation $(n)_k$ for the falling factorial is defined in (5)).

**Use in pedagogy.** Julian Champkin [23] highlights the application of statistics to estimate German tank production during WWII as a “great moment in statistics.” Roger Johnson [27] lists and evaluates several intuitive point estimators for the size of the tank population. Richard Scheaffer et al. [45] propose a hands-on learning activity to illustrate the German tank problem by sampling chips labeled with numbers from 1 to $n$ from a bowl. Inspired by the German tank problem, Arthur Berg [3] orchestrates a classroom-based competition to best estimate the size of a population of a city from a random sample. George Clark, Alex Gonye, and Steven J. Miller [10] explore the use of simulations of tank capturing and linear regression to discover the estimator in (1).

**The Bayesian approach.** Closely related to our pedagogical exploration of the Bayesian approach to the German tank problem, Harry Roberts [41], Michael Höhle, and Leonhard Held [25], Wolfgang Von der Linden, Volker Dose, and Udo Von Toussaint [49], and Simona Cocco, Rémi Monasson, and Francesco Zamponi [11] undertake a Bayesian analysis of the German tank problem and provide an analytical formula for the mean and variance of the posterior distribution of the tank population size under an improper uniform prior distribution. Mark Andrews [1] outlines the Bayesian approach to the German tank problem in a blog post containing code in the R language. William Rosenberg and John Deely [43] outline an empirical Bayesian approach to estimate the number of horses in a race from a sample of numbered horses (the likelihood function here is equivalent to that in the German tank problem). Arthur Berg and Nour Hawila [4] use Bayesian inference for the closely related taxicab problem.

**Generalizations and variants.** Goodman [21, 22] and Clark, Gonye, and Miller [10] pose a variant of the German tank problem in which the initial serial number is not known; i.e., the $n$ tanks are inscribed with serial numbers $\{b+1, \ldots , n+b\}$ with $b$ and $n$ unknown. Lee and Miller [31] generalize the German tank problem to the settings in which the serial numbers belong to a continuum and/or lie in two or more dimensions within a square or circle.

### Overview of the Bayesian Approach to the German Tank Problem

Adopting a Bayesian perspective [6, 15, 46], we treat the (unknown) total number of tanks as a discrete random variable *N* to model our uncertainty about it. A probability mass function of *N* assigns a probability to each possible tank population size *n*. This probability is a measure of our degree of belief, perhaps with some basis in knowledge and data, that the tank population size is *n* [20]. The spread of the mass function of *N* over the integers reflects uncertainty.

The observed serial numbers $(s_1, \ldots , s_k)$ convey information about the tank population size. Hence, the probability mass function of *N* changes after the data $(s_1, \ldots , s_k)$ are collected and considered. That is, *N* has a prior and a posterior probability mass function.

The three inputs to a Bayesian treatment of the German tank problem are as follows:

1. The prior mass function of $N$, which expresses a combination of our subjective beliefs and objective knowledge about the tank population size before we collect and consider the sample of serial numbers.
2. The data, namely the sample of serial numbers $(s_1, \ldots , s_k)$, viewed as realizations of random variables $(S_1, \ldots , S_k)$ owing to the stochasticity of tank-capturing.
3. The likelihood function, giving the probability of the data $(S_1, \ldots , S_k)=(s_1,\ldots ,s_k)$ under each tank population size $N=n$, based on a probabilistic model of the tank-capturing process.

**Figure 1.**
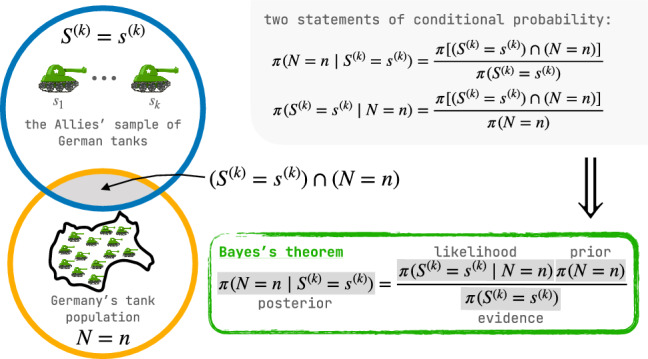
Bayes’s theorem applied to the German tank problem. An Euler diagram [32, 37] represents the two events $S^{(k)}=s^{(k)}$ and $N=n$ with circles. The area of each circle is proportional to the probability of the event, and the area of overlap is proportional to the probability of the intersection $(S^{(k)}=s^{(k)}) \cap (N=n)$ of the events. The Euler diagram rationalizes the two statements of conditional probability in terms of the intersection of the events, which in turn imply Bayes’s theorem [30].

The output of a Bayesian treatment of the German tank problem is the posterior mass function of *N*, conditioned on the data $(s_1, \ldots , s_k)$. The posterior follows from Bayes’s theorem and can be viewed as an update to the prior in light of the data, as illustrated by Figure 1. The posterior mass function of *N* assigns to each possible tank population size *n* a probability according to a compromise between its likelihood, which invokes the probabilistic tank-capturing model to quantify the support lent by the observed serial numbers $(s_1, \ldots , s_k)$ for the hypothesis that the tank population size is *n*, and its prior probability, which quantifies how likely we thought the tank population size was *n* before the serial numbers $(s_1, \ldots , s_k)$ were collected and considered [46]. The posterior mass function of *N* is the raw Bayesian solution to the German tank problem; its spread quantifies our posterior uncertainty about *N*. We may summarize the posterior by reporting its median and a small subset of the integers on which most of the posterior mass sits—a *credible set* that likely contains the tank population size. Furthermore, from the posterior, we can answer questions such as, what is the probability that *N* exceeds some threshold quantity $n^\prime$ that would alter military strategy?

## The Bayesian Approach to the German Tank Problem

We now delve into the details of the Bayesian approach to the German tank problem and illustrate via an example. For reference, the variables are listed in Table 2. We use uppercase letters to represent random variables and lowercase letters to represent their realizations. Throughout, we employ the indicator function associated with a set *A*:

2 $$\begin{aligned} {\mathcal {I}}_{A}(x) = {\left\{ \begin{array}{ll} 1 &{} x \in A, \\ 0 &{} x \notin A . \end{array}\right. } \end{aligned}$$

**Table 2:** List of parameters/variables.

Parameter/variable	$\in$	Description
*n*	${\mathbb {N}}_{\ge 0}$	Size of population of tanks
*k*	${\mathbb {N}}_{>0}$	Number of captured tanks
$s_i$	${\mathbb {N}}_{>0}$	Serial number on captured tank *i*
$s^{(k)}$	${\mathbb {N}}_{>0}^k$	Vector listing the serial numbers on the *k* captured tanks
$m^{(k)}$	${\mathbb {N}}_{>0}$	Maximum serial number among the *k* captured tanks

### The Prior Distribution

We construct the prior probability mass function $\pi _{\text {prior}}(N=n)$ to express a combination of our subjective beliefs and objective knowledge about the total number of tanks *N* before the data $(s_1, \ldots , s_k)$ are collected and considered.

The prior mass function that we impose on *N* depends on the context. If we do not possess prior information about the tank population size, we may adopt the principle of indifference and impose a diffuse prior, e.g., a uniform distribution over a set of feasible tank population sizes. On the other hand, if we possess a rough estimate of the number of tanks from some other source of information or analysis, we may construct a more informative prior that concentrates its mass around this estimate. By definition, a diffuse prior admits more uncertainty (measured, for example, by entropy [35]) about the tank population size than a more informative prior [46].

Thinking ahead about the posterior mass function of *N*, which balances the prior and the likelihood (the latter based on the data), a more informative prior will have a larger impact on the posterior than a diffuse one [46], which “lets the data speak for itself” [15], and generally, as the number of captured tanks *k* increases, we expect the prior to have a smaller impact on the posterior [15] as the data “overwhelms” the prior.

### The Data, Data-Generating Process, and Likelihood Function

**The data.** The data we obtain in the German tank problem is the vector

3 $$\begin{aligned} s^{(k)}:=(s_1,\ldots ,s_k) \end{aligned}$$

of serial numbers inscribed on the *k* captured tanks. We view the data $s^{(k)}$ as a realization of the discrete random vector $S^{(k)}:=(S_1, \ldots , S_k)$. At this point, we are entertaining the possibility that the order in which tanks are captured matters.

**The data-generating process.** The stochastic data-generating process consists in the sequential capture of *k* tanks from a population of *n* tanks, without replacement, and then inspecting their serial numbers to construct $s^{(k)}$. We assume that each tank in the population is equally likely to be captured at each step. Then mathematically, the stochastic data-generating process is a sequential uniform random selection of *k* integers, without replacement, from the set $\{1, \ldots , n\}$.

**The likelihood function.** The likelihood function specifies the probability of the data $S^{(k)}=s^{(k)}$ given each tank population size $N=n$. Each outcome $s^{(k)}$ in the sample space $\Omega _n^{(k)}$ is equally likely, where

4 $$\begin{aligned} \Omega _n^{(k)} := \{ (s_1, \ldots , s_k)_{\ne } : s_i \in \{1, \ldots , n\} \; \text {for all } i \in \{ 1,\ldots , k \} \}, \end{aligned}$$

with $(\cdots )_{\ne }$ meaning that the elements of the vector $(\cdots )$ are unique. The number of outcomes $\bigl \vert \Omega _n^{(k)}\bigr \vert$ in the sample space is the number of distinct ordered arrangements of *k* distinct integers from the set $\{1,\ldots ,n\}$, given by the falling factorial:

5 $$\begin{aligned} (n)_k:= n(n-1)\cdots (n-k+1) = \frac{n!}{(n-k)!}. \end{aligned}$$

Under the data-generating process, then, the probability of observing data $S^{(k)}=s^{(k)}$ given the tank population size $N=n$ is the uniform distribution:

6 $$\begin{aligned} \pi _{\text {likelihood}}\left(S^{(k)}=s^{(k)} \mid N=n\right)= \dfrac{1}{(n)_k} {\mathcal {I}}_{\Omega _n^{(k)}}\left( s^{(k)}\right) . \end{aligned}$$

**Interpretation.** The likelihood quantifies the support provided by the serial numbers on the *k* captured tanks in $s^{(k)}$, when compared with our probabilistic model of the tank-capturing process, for the hypothesis that the tank population size is *n* [46]. We view $\pi _{\text {likelihood}}(S^{(k)}=s^{(k)} \mid N=n)$ as a function of *n*, since in practice we possess the data $s^{(k)}$ but not *n*.

**The likelihood as a sequence of events.** Alternatively, we may arrive at (6) from a perspective of sequential events $S_1=s_1, S_2=s_2, \ldots , S_k=s_k$. First, the probability of a given serial number on the $i$th captured tank, conditioned on the tank population size and the serial numbers on the previously captured tanks, is the uniform distribution

7 $$\begin{aligned}{} & {} \pi (S_i=s_i \mid N=n, S_1=s_1, \ldots , S_{i-1}=s_{i-1})\nonumber \\{} & {} \quad =\dfrac{1}{n-i+1} {\mathcal {I}}_{ \{1,\ldots ,n\} \setminus \{s_1, \ldots , s_{i-1}\}}(s_i), \end{aligned}$$

since there are $n-(i-1)$ tanks to choose from, uniformly and randomly. By the chain rule of probability [29], the joint probability is

8 $$\begin{aligned}{} & {} \pi _{\text {likelihood}}(S_1=s_1, \ldots , S_k=s_k \mid N=n) \nonumber \\{} & {} \quad = \displaystyle \prod _{i=1}^k \pi (S_i=s_i \mid N=n, S_1=s_1,\ldots ,S_{i-1}=s_{i-1}), \end{aligned}$$

which gives (6) after simplifying the product of indicator functions.

**The likelihood function in terms of the maximum observed serial number.** We will find out below that only two independent features of the data $(s_1, \ldots , s_k)$ provide information about the tank population size $N$: its size, $k$, and the maximum observed serial number

9 $$\begin{aligned} m^{(k)} = \max _{i \in \{1, \ldots , k\}} s_i . \end{aligned}$$

Thus, we also write a different likelihood: the probability $\pi _{\text {likelihood}}(M^{(k)}=m^{(k)} \mid N=n)$ of observing a maximum serial number $m^{(k)}$ given the tank population size $N=n$.

Because each outcome $s^{(k)}\in \Omega _n^{(k)}$ is equally likely, $\pi _{\text {likelihood}}(M^{(k)}=m^{(k)} \mid N=n)$ is the fraction of the sample space $\Omega _n^{(k)}$ in which the maximum serial number is $m^{(k)}$. To count the outcomes $s^{(k)}\in \Omega _n^{(k)}$ where the maximum serial number is $m^{(k)}$, consider that one of the *k* captured tanks has serial number $m^{(k)}$ and the remaining $k-1$ tanks have a serial number in $\{1, \ldots , m^{(k)}-1\}$. For each of the *k* possible positions of the maximum serial number in the vector $s^{(k)}$, there are $(m^{(k)}-1)_{k-1}$ distinct outcomes specifying the other $k-1$ entries. Thus

10 $$\begin{aligned}{} & {} \pi _{\text {likelihood}}(M^{(k)}=m^{(k)} \mid N=n) \nonumber \\{} & {} \quad = \dfrac{k(m^{(k)}-1)_{k-1}}{(n)_k} {\mathcal {I}}_{\{k,\ldots ,n\}}(m^{(k)}). \end{aligned}$$

### The Posterior Distribution

The posterior probability mass function of *N* assigns a probability to each possible tank population size *n* in consideration of its consistency with the data $(s_1, \ldots , s_k)$, according to the likelihood in (6), and our prior beliefs/knowledge encoded in $\pi _{\text {prior}}(N=n)$.

The posterior distribution is a conditional distribution related to the likelihood and prior mass functions by Bayes’s theorem [30] (see Figure 1):

11 $$\begin{aligned}{} & {} \pi _{\text {posterior}}(N=n \mid S^{(k)}=s^{(k)}) \nonumber \\{} & {} \quad = \frac{\pi _{\text {likelihood}}(S^{(k)}=s^{(k)} \mid N=n) \pi _{\text {prior}}(N=n)}{\pi _{\text {evidence}}(S^{(k)}=s^{(k)})}. \end{aligned}$$

The denominator, the *evidence* [30], is the probability of the data $s^{(k)}$:

12 $$\begin{aligned}{} & {} \pi _{\text {evidence}}(S^{(k)}=s^{(k)}) \nonumber \\{} & {} \quad = \displaystyle \sum _{n^\prime =0}^\infty \pi _{\text {likelihood}}\left(S^{(k)}=s^{(k)} \mid N=n^\prime \right) \pi _{\text {prior}}(N=n^\prime ). \end{aligned}$$

We view $\pi _{\text {posterior}}\left( N=n \mid S^{(k)}=s^{(k)}\right)$ as a probability mass function of *N*, since in practice, we have $s^{(k)}$. Then $\pi _{\text {evidence}}\left( S^{(k)}=s^{(k)}\right)$, which is independent of *n*, is just a normalizing factor for the numerator in (11).

In interpreting (11), the prior mass function of *N* is updated, in light of the data $(s_1, \ldots , s_k)$, to yield the posterior mass function of *N*. The posterior probability that $N=n$ is proportional to the product of the likelihood at and prior probability of $N=n$, giving a compromise between the likelihood and prior.

We simplify the posterior mass function of *N* in (11) by substituting (6), restricting the sum in (12) to tank population sizes where the likelihood is nonzero, and noting that the only two features of the data $(s_1, \ldots , s_k)$ that appear are its size *k* and the maximum serial number $m^{(k)}$:

13 $$\begin{aligned}&\pi _{\text {posterior}}(N=n \mid S^{(k)}=s^{(k)}) \nonumber \\&\quad = \pi _{\text {posterior}}(N=n \mid M^{(k)}=m^{(k)}) \nonumber \\&\quad = \frac{ \displaystyle (n)_k^{-1} \pi _{\text {prior}}(N=n) }{ \displaystyle \sum _{n^\prime =m^{(k)}}^\infty (n^\prime )_{k}^{-1} \pi _{\text {prior}}(N=n^\prime ) } {\mathcal {I}}_{\{m^{(k)}, m^{(k)}+1,\ldots \}}(n). \end{aligned}$$

Note, we may arrive at (13) through (10) as well.

**Interpretation.** The posterior probability mass function of *N* in (13) assigns a probability to each tank population size *n* in consideration of the serial numbers $(s_1, \ldots , s_k)$ observed on the captured tanks, our probabilistic model of the tank-capturing process, and our prior beliefs and knowledge about the tank population size expressed in the prior mass function of *N*. The spread (measured, e.g., by entropy) of the posterior mass function of *N* reflects remaining epistemic (reducible with more data) [17, 47] uncertainty about the tank population size.

**A remark on “uncertainty.”** The source of posterior uncertainty is a lack of complete data: we have not captured all of the tanks^3^ and observed their serial numbers to be certain of the tank population size. In practice, an additional source of posterior uncertainty about the tank population size is the possible inadequacy of the model of the tank-capturing process (uniform sampling) in (6). That is, selection bias could be present in the tank-capturing process. Our analysis here neglects this source of uncertainty.

**Summarizing the posterior mass function of** $\varvec{N}$. We may summarize the posterior mass function of $N$ with a point estimate of the tank population size and a credible subset of the integers that contains the tank population size with a high probability.^4^ A suitable point estimate of the tank population size is a median of the posterior mass function of $N$; by definition, the posterior probability that the tank population size is greater (less) than or equal to a median is at least 0.5. A suitable credible subset, which entertains multiple tank population sizes, is the $a$-high-mass subset [26]

14 $$\begin{aligned} {\mathcal {H}}_a:= \left\{ n^\prime : \pi _{\text {posterior}}\left( N=n^\prime \mid M^{(k)}=m^{(k)}\right) \ge \pi _a\right\} , \end{aligned}$$

where $\pi _a$ is the largest mass to satisfy

15 $$\begin{aligned} \pi _{\text {posterior}}\left( N \in {\mathcal {H}}_a \mid M^{(k)}=m^{(k)}\right) \ge 1 - a. \end{aligned}$$

In words, the *a*-high-mass subset ${\mathcal {H}}_a$ is the smallest that contains at least a fraction $1-a$ of the posterior mass of *N* and ensures that every tank population size belonging to it is more probable than any population size outside of it.

**Querying the posterior distribution.** We may find the posterior probability that the tank population size belongs to any set of interest by summing the posterior mass over it; e.g., the probability that the tank population size exceeds some number $n^\prime$ is

16 $$\begin{aligned}{} & {} \pi _{\text {posterior}}\left( N> n^\prime \mid M^{(k)}=m^{(k)}\right) \nonumber \\{} & {} \quad = \sum _{n=n^\prime +1}^\infty \pi _{\text {posterior}}\left( N=n \mid M^{(k)}=m^{(k)}\right) . \end{aligned}$$

## An Example

We illustrate the Bayesian approach to the German tank problem through an example.

**Figure 2.**
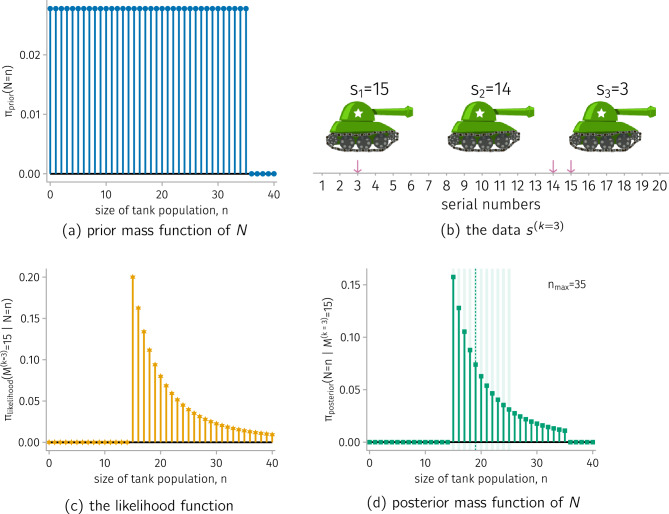
A Bayesian approach to the German tank problem. (a) The prior mass function. (b) The data $s^{(3)}$, with maximum observed serial number $m^{(3)}=15$. (c) The likelihood function associated with the data $s^{(3)}$. (d) The posterior mass function of *N*; ${\mathcal {H}}_{0.2}$ is highlighted, and the median is marked with a vertical dashed line.

**The prior probability mass function of** $\textbf{N}$. Suppose we have an upper bound $n_{\max }$ for the possible number of tanks, based on, e.g., the supply of some raw material needed for tank production, but no other information. Then we may impose a diffuse prior, a uniform prior probability mass function

17 $$\begin{aligned} \pi _{\text {prior}}(N=n) = \dfrac{1}{n_{\max }+1} {\mathcal {I}}_{ \{0, \ldots , n_{\max }\}}(n). \end{aligned}$$

This prior mass function expresses that in the absence of any data $(s_1, \ldots , s_k)$ (i.e., no serial numbers, and not even *k*), we believe that the total number of tanks *N* is equally likely to be any value in $\{0, \ldots , n_{\max }\}$. Particularly, suppose $n_{\max} =35$. Figure 2a visualizes $\pi _{\text {prior}}(N=n)$.

**The data** $({\varvec{s}}_{\textbf{1}}, \ldots , {\varvec{s}}_{\varvec{k}})$ **and the likelihood function.** Now suppose we capture $k=3$ tanks, with serial numbers $s^{(3)}=(15, 14, 3)$. See Figure 2b. So the maximum observed serial number is $m^{(3)}=15$. The likelihood function $\pi _{\text {likelihood}}(M^{(3)}=15 \mid N=n)$ in (10) is displayed in Figure 2c. Note that the likelihood function is maximal at $n=m^{(3)}=15$ and decreases monotonically.

**The posterior probability mass function of** $\varvec{N}$. Under the uniform prior in (17), the posterior probability mass function of $N$ in (13) becomes

18 $$\begin{aligned}{} & {} \pi _{\text {posterior}}\left( N=n \mid M^{(k)}=m^{(k)}\right) \nonumber \\{} & {} \quad = \dfrac{(n)_k^{-1} }{ \displaystyle \sum _{ n^\prime = m^{(k)}}^{n_{\max }} (n^\prime )_k^{-1} } {\mathcal {I}}_{ \{m^{(k)}, m^{(k)}+1, \ldots , n_{\max }\} }(n). \end{aligned}$$

Figure 2d visualizes the posterior probability mass function of *N* for the data $s^{(3)}$ in Figure 2b and the prior in (17) ($n_{\max }=35$).

**Summarizing the posterior.** The posterior mass function of $N$ has median 19 and high-mass credible subset ${\mathcal {H}}_{0.2}=\{15, \ldots , 25\}$ (highlighted in Figure 2d). For what it’s worth, the data in Figure 2b were generated from a tank population size of $n=20$ (explaining the choice of scale in Figure 2b).

**Querying the posterior.** Suppose our military strategy would change if the size of the tank population were to exceed 30. From the posterior distribution of $N$, we calculate $\pi _{\text {posterior}}(N>30 \mid M^{(3)}=15)\approx 0.066$.

**Figure 3.**
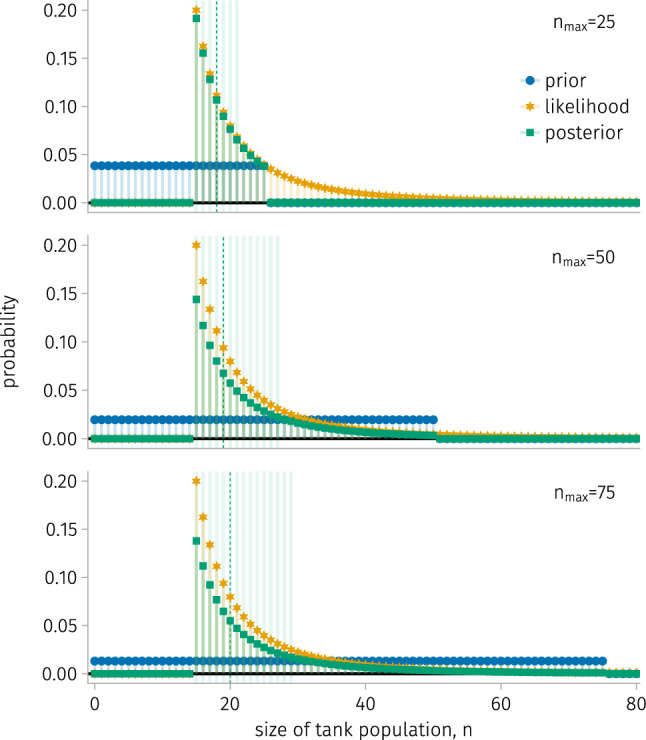
Evaluating the sensitivity of the posterior mass function of *N* to the upper bound $n_{\max }$ imposed by the prior mass function of *N*.

**Sensitivity of the posterior to the prior.** Because of the subjectivity involved in constructing the prior, checking the sensitivity of the posterior to the prior is good practice [46]. Figure 3 shows how the posterior mass function of $N$ changes with the upper bound on the tank population $n_{\max }$ that we impose via the prior mass function of $N$ in (17). For example, under $n_{\max } =75$, the high-mass subset ${\mathcal {H}}_{0.2}$ expands to $\{15, \ldots , 29\}$.

**Figure 4.**
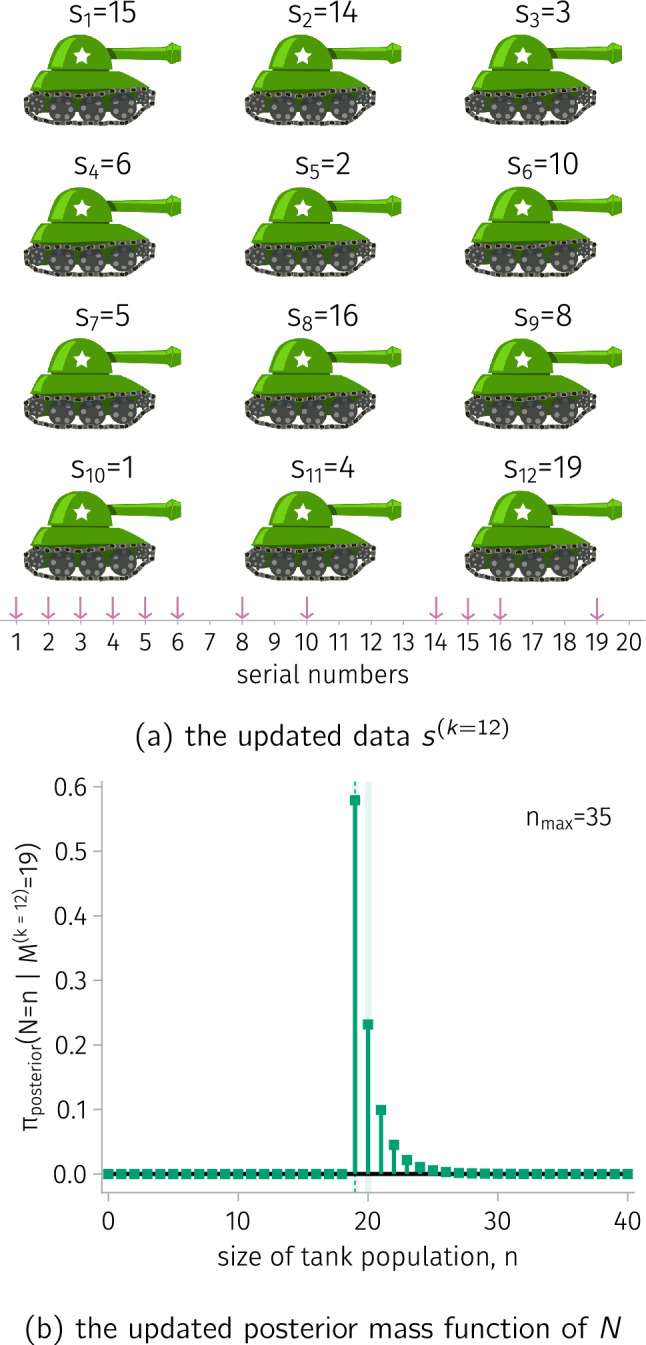
The posterior distribution of *N* after we capture more tanks. (a) We capture an additional nine tanks. (b) The updated posterior mass function of *N*.

**Capturing more tanks.** Suppose we capture an additional nine tanks and rerun the Bayesian analysis. Figure 4 shows the updated posterior mass function of $N$. The high-mass credible subset ${\mathcal {H}}_{0.2}$ shrinks considerably, to $\{19, 20\}$. This shows how more data—a larger number $k$ of tanks captured—generally reduces our uncertainty about the tank population size.

## Simulations to Investigate the Behavior of the Posterior of *N* Under a Known Population Size

We now investigate how, on average, over the stochastic outcomes of the tank-capturing process for a fixed tank population size, the posterior distribution $\pi _{\text {posterior}}\left( N=n \mid S^{(k)}=s^{(k)}\right)$ depends on the number *k* of tanks captured and the maximum $n_{\max }$ of the support of the uniform prior.

**Figure 5.**
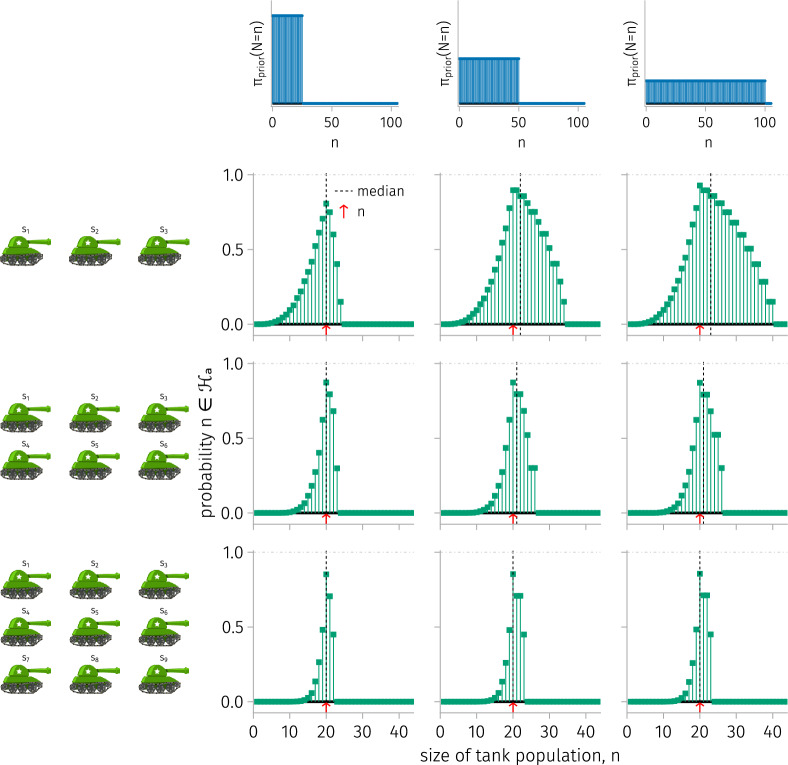
The average high-mass subset and median of the median of the posterior over tank-capturing under a fixed tank population size. Rows: different numbers *k* of tanks captured (left). Columns: different maxima of tank population sizes entertained by the uniform prior $n_{\max }$ (top). For a particular $(k, n_{\max })$, the stem plots show the probability of each tank population size belonging to the high-mass subset ${\mathcal {H}}_{a=0.2}$ of the posterior. The vertical dashed line shows the median of the median of the posterior. The red arrow shows the true tank population size, 20.

For a given *k* and $n_{\max }$, we conduct 50,000 simulations, in each of which *k* random tanks are captured from a population of $n=20$ tanks, giving data $s^{(k)}$; computing the posterior mass function $\pi _{\text {posterior}}\left( N=n \mid S^{(k)}=s^{(k)}\right)$; then finding the high-mass subset ${\mathcal {H}}_a$ ($a=0.2$) and median of the posterior. Figure 5 displays $(1, \text {stems})$, the probability of each tank population size *n* belonging to ${\mathcal {H}}_a$, and $(2, \text {vertical line})$, the median of the median of the posterior, for $(k, n_{\max })\in \{3, 6, 9\} \times \{25, 50, 100\}$.

As *k* increases, the high-mass subset ${\mathcal {H}}_a$ tends to be less sensitive to $n_{\max }$, since the data overrides the prior, and to shrink, since uncertainty decreases with a larger sample. As $n_{\max }$ increases, larger population sizes become more likely to be included in ${\mathcal {H}}_a$. The median of the median of the posterior matches the true tank population size of 20 when $n_{\max }=25$ or $k=9$. For $k\in \{3, 6\}$, the larger $n_{\max }$ values pull the median above the true tank population size.

## Discussion

**Selection bias.** A strict assumption in the textbook-friendly German tank problem, which enables us to estimate the size of the population of tanks from a random sample of their (sequential) serial numbers, is that sampling is uniform. To check consistency of the sample with this model of the tank-capturing process, Goodman [22] demonstrates a test of the hypothesis that the sample of serial numbers is from a uniform distribution. Interesting extensions of the textbook German tank problem could involve modeling selection bias in the tank-capturing process. For example, such bias could arise hypothetically if older tanks with smaller serial numbers were more likely to be deployed in the fronts opened earlier in the war, where capturing tanks is more difficult than at less fortified fronts opened more recently. Selection bias could also manifest in clusters in the observed serial numbers.

**The German tank problem in other contexts.** The Bayesian probability theory used to solve the German tank problem applies (perhaps with modification) to many other contexts in which we wish to estimate the size of some finite hidden set [9], such as the number of taxicabs in a city [19, 23], racing cars on a track [48], accounts at a bank [25], pieces of furniture purchased by a university [22], aircraft operations at an airport [34], cases in court [50], or electronic devices produced by a company [2]. And also the extent of leaked classified government communications [18], the time needed to complete a project deadline [16], the time-coverage of historical records of extreme events like floods [39], the length of a short-tandem repeat allele [51], the size of a social network [28], the lifetime of a flower of a plant [38], or the duration of existence of a species [42]. In addition, mark and recapture methods in ecology to estimate the size of an animal population [8, 36] are tangentially related to the German tank problem.

**The practice of inscribing sequential serial numbers on military equipment.** Germany adopted the practice of marking their military equipment with serial numbers and codes to trace the equipment/parts/components back to the manufacturer. However, the sequential nature of those serial numbers was exploited by the Allies to estimate their armament production. To reduce vulnerability to serial number analysis for estimating production while maintaining the advantages of tracing equipment back to the manufacturer, serial numbers and codes could instead be encrypted [14] or obfuscated, for instance by the method known as chaffing [40].

## Data Availability

The Julia [5] code to reproduce all of our visualizations drawn using Makie.jl [12] is available on Github at https://www.github.com/SimonEnsemble/the_German_tank_problem.

## Footnotes

1 For example, captured records from tank repair depots listed serial numbers of the chassis and engine of repaired tanks, and records from divisional headquarters listed chassis serial numbers of tanks held by a specific unit.

2 Gearboxes on captured tanks, for example, were inscribed with serial numbers belonging to an unbroken sequence. Chassis serial numbers, on the other hand, were broken into blocks to distinguish models/designs, leaving gaps between the serial numbers assigned to them.

3 Certainly, $k<n$ if there are gaps in the observed serial numbers $(s_1, \ldots , s_k)$. Even if there are no gaps in $(s_1, \ldots , s_k)$, we cannot be certain we have captured the tank with the largest serial number.

4 Under our assumptions embedded in the likelihood and prior mass functions.
